# Complete chloroplast genome of *Prunus emarginata* and its implications for the phylogenetic position within *Prunus sensu lato* (Rosaceae)

**DOI:** 10.1080/23802359.2019.1674212

**Published:** 2019-10-09

**Authors:** Lu-Xi Chen, Kevin R. Brown, Xian-Gui Yi, Zhong-Shuai Sun

**Affiliations:** a Zhejiang Provincial Key Laboratory of Plant Evolutionary Ecology and Conservation, Taizhou University, Taizhou, China;; b KR Brown and Associates, Victoria, Canada;; c Co-Innovation Center for the Sustainable Forestry in Southern China, College of Biology and Environment, Nanjing Forestry University, Nanjing, China

**Keywords:** *Prunus emarginata*, *Prunus sensu lato*, chloroplast genome, phylogenomics

## Abstract

*Prunus emarginata* is a species of Prunus native to western North America.We determined the first complete chloroplast genome of *P. emarginata* using genome skimming approach. The cp genome was 157,458 bp long, with a large single-copy region (LSC) of 85,567 bp and a small single-copy region (SSC) of 19,121 bp separated by a pair of inverted repeats (IRs) of 26,385 bp. It encodes 129 genes, including 84 protein-coding genes, 37 tRNA genes, and 8 ribosomal RNA genes. We also reconstructed the phylogeny of *Prunus sensu lato* using maximum likelihood (ML) method, including our data and previously reported cp genomes of related taxa. The phylogenetic analysis indicated that *P. emarginata* is closely related with *Prunus subhirtella* var. *subhirtella* and *Prunus yedoensis*.

*Prunus emarginata* (Douglas) Eaton, bitter cherry, is a species of Prunus native to western North America, extending from British Columbia south to Baja California, and east as far as western Wyoming and New Mexico (Taylor and Taylor 1981; Rohrer 2014). The classification of the *Prunus sensu lato* (Rosaceae) has long been problematic; phylogenetic studies using a limited set of markers have often not been able to fully resolve relationships within this genus, indicating that a higher number of molecular characters are required for an improved understanding of relationships within this group (Shi et al. 2013; Chin et al. 2014). By taking advantages of next-generation sequencing technologies that efficiently provide the chloroplast (cp) genomic resources of our interested species, we can rapidly access the abundant genetic information for phylogenetic research and conservation genetics (Liu et al. 2017, 2018). Therefore, we sequenced the whole chloroplast genome of *P. emarginata* to elucidate its phylogenetic relationship with other *Prunus sensu lato*.

Total genomic DNA was extracted from silica-dried leaves collected from Bowser on the east coast of Vancouver Island (British Columbia, Canada) using a modified CTAB method (Doyle and Doyle 1987). The voucher specimen (Brown1808001) was collected and deposited in the Herbarium of Taizhou University. DNA libraries preparation and pair-end 125 bp read length sequencing were performed on the Illumina HiSeq 2500 platform. About 7.68 Gb of raw data were trimmed and assembled into contigs using CLC Genomics Workbench 8. All the contigs were then mapped to the reference cp genome of *Prunus speciosa* (Koidz.) Nakai (MH998233; Sun et al. 2019) using BLAST (NCBI BLAST v2.2.31) search and the draft *cp* genome of *P. speciosa* was constructed by connecting overlapping terminal sequences in Geneious R11 software (Biomatters Ltd., Auckland, New Zealand). Gene annotation was performed via the online programme Dual Organellar Genome Annotator (DOGMA; Wyman et al. 2004).

The complete cp genome of *P. emarginata* (GenBank accession MN389436) was 157,458 bp long consisting of a pair of inverted repeat regions (IRs with 26,385 bp) divided by two single-copy regions (LSC with 85,567 bp; SSC with 19,121 bp). The overall GC contents of the total length, LSC, SSC, and IR regions were 36.7%, 34.5%, 30.1% and 42.5%, respectively. The genome contained a total of 129 genes, including 84 protein-coding genes, 37 tRNA genes and 8 rRNA genes.

To determine the phylogenetic position of newly sequenced *P. emarginata*, phylogenetic analysis was conducted along with 18 representative *Prunus sensu lato* species and two outgroup taxa. We reconstructed a phylogeny employing the GTR + G model and 1000 bootstrap replicates under the maximum-likelihood (ML) inference in RAxML-HPC v.8.2.10 on the CIPRES cluster (Miller et al. 2010). The ML tree (Figure 1) was consistent with the most recent phylogenetic study on *Prunus sensu lato* (Shi et al. 2013; Chin et al. 2014). *P. emarginata* exhibited the closest relationship with *Prunus subhirtella var. subhirtella* and *Prunus* × *yedoensis*.

**Figure 1.**
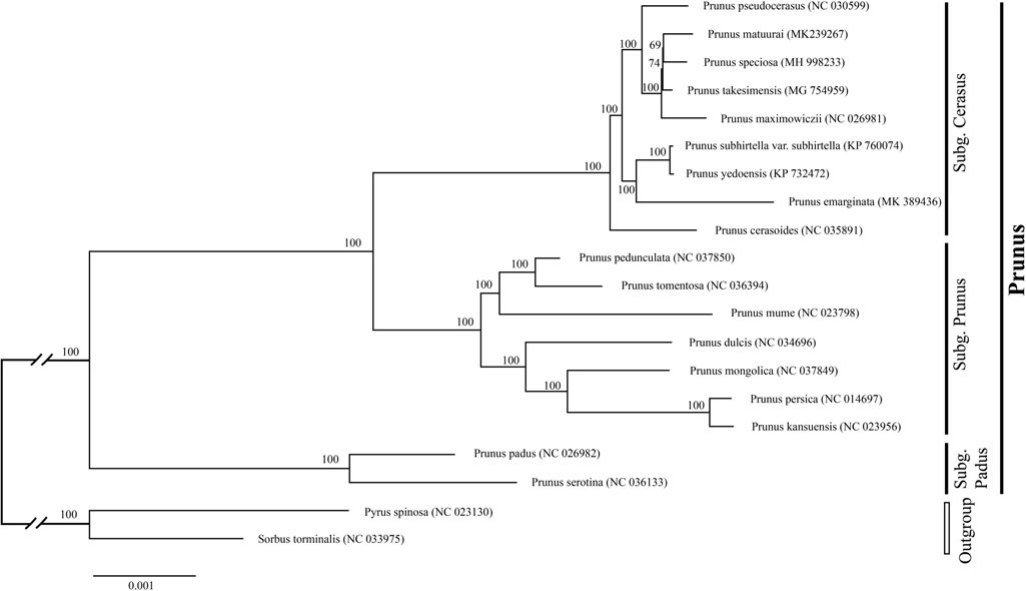
Phylogenetic tree reconstruction of 18 taxa of *Prunus* and two outgroups using ML method. Relative branch lengths are indicated. Numbers near the nodes represent ML bootstrap value. The scientific names of some species are debated.
